# Knowledge and practice of prevention of mother-to-child transmission of HIV among traditional birth attendants in Lagos State, Nigeria

**Published:** 2010-04-29

**Authors:** Mobolanle Balogun, Kofo Odeyemi

**Affiliations:** 1 Department of Community Health and Primary Care, College of Medicine University of Lagos, Nigeria

**Keywords:** HIV, AIDS, PMTCT, knowledge, practice, traditional birth attendants, Nigeria

## Abstract

**Background:**

Traditional birth attendants (TBAs) assist most deliveries in Nigeria. Knowing and understanding all issues surrounding HIV/AIDS and Prevention of Mother-To-Child Transmission of HIV (PMTCT) can help them to protect themselves and others. This study aimed to assess the knowledge and practice of PMTCT amongst TBAs in Lagos, Nigeria.

**Methods:**

This was a cross-sectional survey. Multistage sampling method was used to select 108 registered TBAs in 2 local governments areas who were interviewed using a pre-tested questionnaire.

**Results:**

All the respondents were aware of HIV but their awareness of PMTCT specifically was not as high. Only 8.3% of the respondents had good level of knowledge about HIV and PMTCT and up to 13% of them claimed to be able to cure HIV using native remedies. The practices of HIV counseling of patients and referral of patients for HIV testing were low and higher levels of knowledge positively influenced these practices significantly (p < 0.05). They were also deficient in certain measures to prevent infection of patients and themselves.

**Conclusion:**

Most of the TBAs did not have adequate knowledge and practice of PMTCT illustrating the need for periodic PMTCT training for TBAs.

## Background

Nigeria, with an estimated national HIV prevalence of 4.6 %, has the third largest number of people living with HIV/AIDS in the world and the highest number of HIV-infected adults in West Africa. At the current level of prevalence, it is estimated that 2.95 million people in Nigeria are currently infected with HIV. [1-2] Heterosexual transmission accounts for nearly 80% of all infections, about 10% of HIV infections are transmitted through mother-to-child transmission (MTCT), while another 10% is transmitted by the use of unsterilized needles and surgical implements, infected blood and blood products. [2-3] In 2009 an estimated 1.72 million women 15-49 years old and 278,000 children in Nigeria were living with HIV. [2] More than 90% of the infections among children occur through MTCT, the rate of which is affected by many factors, including high viral load, mode of delivery, prolonged rupture of membranes, prematurity and breastfeeding [4].

One of the goals of the June 2001 Declaration of Commitment of the United Nations General Assembly Special Session on HIV/AIDS (UNGASS) is to reduce the proportion of infants infected with HIV by 20% by 2005 and 50% by 2010. The national goal for PMTCT as contained in the AIDS Policy for Nigeria is to reduce the transmission of HIV through MTCT by 50% by the year 2010 and to increase access to quality voluntary confidential counseling and testing services by 50% by the same year. [4] This is ultimately more achievable if all PHC providers are integrated into the nation’s PMTCT programme. An effective PHC service is already established throughout Nigeria. Since October 1988, PHC providers are recognised as including TBAs, voluntary village head workers, junior and senior community health extension workers and community health officers (CHOs). [5] However, PMTCT services in Nigeria are still concentrated at tertiary level, with little collaboration with PHC centers or community support services. [6]

As many as 60% of children born in Nigeria are delivered by TBAs [5] who speak the local languages, allow traditional birthing practices, and often have the trust and respect of the community. Both rural and urban women may seek care with TBAs because they share the same cultural codes and have similar socio-economic characteristics. [7-9].

Delivery involves exposure to blood and body fluids hence traditional birth attendants should be able to protect mothers, children and themselves. Knowing and understanding all issues surrounding HIV/AIDS, modes of transmission, signs and symptoms and infection control can help health workers and all traditional birth attendants to protect themselves and others. Some tasks that TBAs could perform to help prevent perinatal transmission of HIV include dissemination of information about how HIV can be transmitted between mother and child and explanation of effective strategies to prevent such transmission; identification of pregnant women in their communities and facilitation of their use of available antenatal and maternity care; ensuring that pregnant women and their partners are routinely offered HIV counseling and testing and that their uptake of this is facilitated; reinforcement of health messages, including the importance of improved nutrition during pregnancy; supervision of directly observed treatment of mother and infant with nevirapine; and offering advice on reducing the risk of HIV transmission to women and their partners [10].

This study was designed to assess the knowledge and practice of PMTCT among TBAs in Lagos state, Nigeria.

## Methods

### Study area

This study was conducted in Lagos state in May 2008. Lagos State, called the “State of Excellence and Aquatic Splendour” is one of the 36 states of the Federal Republic of Nigeria. Although Lagos state is the smallest state in the country in terms of landmass, the National Population Commission (NPC) put the 2006 provisional census figure for the state as 9,013,534 out of the total national figure of 140 million. Lagos state is characterized by overcrowded living conditions and increased proliferation of low-income earners as a result of rural-urban migration. The state is

made up of 5 administrative divisions, namely Lagos, Ikeja, Ikorodu, Epe and Badagry. These divisions are further divided into 20 local government areas.

Sampling and data collection

The design was a cross-sectional study. Multistage sampling was used to select the target populations as follows: First, two divisions, Badagry and Ikeja, were selected from the five administrative divisions in Lagos State using simple random sampling by balloting. Second, one local government area was selected from each selected division using simple random sampling (balloting) and they were Ajeromi-Ifelodun and Mushin LGAs. Lastly, all 108 TBAs registered with the two LGAs were recruited for the study.

Two trained field interviewers collected data using a pre-tested, structured 42-item questionnaire. Information was collected about the sociodemographic characteristics of respondents, occupational history, knowledge about HIV and PMTCT, practice of PMTCT and their use of universal precautions.

### Ethical consideration

The study proposal was approved by the Research and Ethics Committee of the Lagos University Teaching Hospital (LUTH). Informed consent was obtained from the TBAs prior to the administration of questionnaires.

### Data analysis

Data collected was entered and analyzed using EPI info 2007 software statistical package (Windows version 3.4.1). Significant differences were evaluated using Chi-squared test, Fisher’s exact test and t-statistic where appropriate. The p-value of <0.05 was taken as statistically significant.

Each respondent’s level of knowledge was determined with a scoring system developed by the researcher. Twenty-four (24) questions on knowledge of HIV and PMTCT were scored, each right answer attracting one point. Those who scored less than 40% (i.e. 0 to 9 points) were classified as having poor knowledge; those who scored between 40% and 60% (i.e. 10 to 14 points) were classified as having fair knowledge and those who scored over 60% were classified as having good knowledge.

## Results

Most (59.3%) of the respondents were females and the majority (98.1%) of them were aged 30 years and above with a combined mean age of

47.1 years ± 12.1 SD. The respondents were mostly married (80.6%), Muslim (58.3%), of Yoruba ethnicity (95.4%) and close to one-half of them had secondary education (Table 1).

The highest proportion (41.7%) of the TBAs had worked for 1 to 10 years and had been trained on the job by their fathers and the mean duration of training was 9.2 years ± 5.7 SD. The mean number of deliveries they took per month was 6.2 ± 4.1 SD (Table 2).

All of the TBAs were aware of HIV/AIDS while 76.9% of them had heard of PMTCT. The most common source of information on HIV was from health workers (78.7%) followed by electronic media (30.6%) and other TBAs (7.4%). One hundred and six (98.1%) respondents knew that HIV is the virus that causes infection/AIDS disease.

The modes of transmission of HIV commonly known to the TBAs were sexual intercourse (78.7%) and contaminated sharps (77.8%). Only 20.4% of them spontaneously mentioned HIV transmission from infected mother to child as a possible mode of transmission. Over a quarter (26.9%) gave some misconceived responses such as kissing, sharing cups/utensils and sharing toilets as possible modes of transmission of HIV. When probed specifically about HIV transmission from an infected mother to child, the majority (90.7%) of them agreed that an infected mother can transmit HIV to her child. However, while most (62%) of them recognized breastfeeding as a possible period of transmission, only 31.5% of them mentioned delivery as a possible period of transmission (Table 3). Less than half (34.3%) of the TBAs correctly knew that HIV couldn’t be cured while 13% believed they could cure HIV.

The commonest ways of preventing mother-to-child transmission of HIV known to the respondents were avoidance of breastfeeding (37%) and HIV counseling and testing (27.8%). Close to a quarter (22.2%) mentioned use of native methods as a way to prevent mother-to-child transmission of HIV, specifically through the use of agbo (a native concoction). Only 55.6% had heard of infant feeding options for PMTCT; they were mostly aware of infant formula as an option (50%) and a small proportion (3.7%) mentioned native concoction agbo as an option. When the knowledge of the respondents was scored, over half of the respondents (58.3%) had a poor level of knowledge of HIV and PMTCT. Only 8.3% of them had good knowledge. The female TBAs had significantly better knowledge than their male counterparts (p < 0.05).

Regarding their practice of PMTCT, only 32.4% of respondents counseled all their patients about HIV and only 42.6% of them referred all their patients for HIV testing. There was a statistically significant association between level of knowledge and the practice of respondents (p < 0.05) as higher proportions of respondents with fair and good levels of knowledge claimed to counsel their patients and refer them for HIV testing (Table 4). Only 37 TBAs (34.3%) reported checking to know the HIV status of all their pregnant patients however majority (93.5%) had no objection to referring HIV positive patients to the hospital.

**Figure 1:**
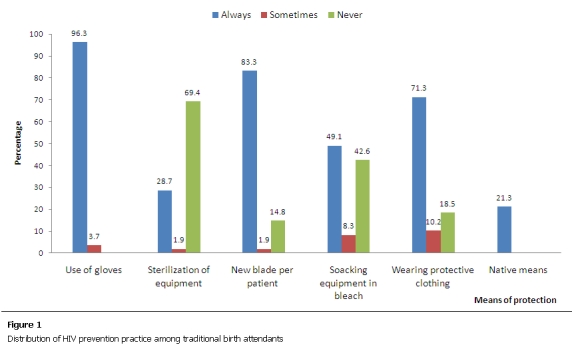
Distribution of HIV prevention pratice among traditional birth attendants

Regarding their HIV prevention practices, hand gloves were always used by 96.3% of the respondents but 16 (14.8%) of them failed to use new blades per patients and 69.4% were not in the habit of sterilizing their equipments. Twenty-three (21.3%) of them mentioned the ingestion of local concoction, “agbo” by the respondents as well as their patients as means of protection against HIV (Figure 1).

**Table 1:** Socio-demographic characteristics of respondents

**Characteristics**	**Frequency**	**Percentage**
**Age group (years)**		
20-29	2	1.9
30-39	31	28.7
40-49	39	36.1
50-59	15	13.9
60+	21	19.4
**Sex**		
Female	64	59.3
Male	44	40.7
Marital status		
Married	87	80.6
Separated	7	6.5
Single	4	3.7
Widowed	10	9.3
Education		
None	29	26.9
Primary	22	20.4
Secondary	53	49.1
Tertiary	4	3.7
Religion		
Christianity	42	38.9
Islam	63	58.3
Traditional	3	2.8
Ethnicity		
Yoruba	103	95.4
Igbo	5	4.6

**Table 2:** Occupational history of respondents

**Variables**	**Frequency**	**Percentage**
**Duration of practice (years)**		
1-10	45	41.7
11-20	32	29.6
21-30	14	13.0
>30	17	15.7
**Source of training**		
Father	45	41.7
Mother	9	8.3
Other relatives	16	14.8
Other TBAs	38	35.2
**Duration of training (years)**		
1-10	76	70.4
>10	32	29.6
**Place of abode**		
Within LGA	105	97.2
Outside LGA	3	2.8
**Deliveries/month**		
1-5	62	57.4
6-10	36	33.3
>10	10	9.3
Other work activities*		
Family planning	10	9.3
Scarifications/incisions	11	10.2
Circumcisions	10	
Infertility treatment	20	1.51

* Multiple responses possible

**Table 3:** Respondents’ knowledge of modes of transmission of HIV and MTCT of HIV

	**Frequency**	**Percentage**
*Modes of transmission		
Sexual Intercourse	85	78.7
Contaminated sharps	84	77.8
Infected mother to child	22	20.4
Blood transfusion	40	37.0
Misconceived responses	29	26.9
**Possibility of MTCT of HIV**		
Yes	98	90.7
No	8	7.4
Don’t know	2	1.9
*Period of MTCT of HIV		
During pregnancy	44	40.7
During delivery	34	31.5
While breastfeeding	67	62.0

* Multiple responses possible

**Table 4:** Association between level of knowledge and practice of respondents

Level of knowledge	HIV counseling of patients				Referral of patients for HIV testing			
	Counsels some/all		Does not counsel		Total	Refers some/all		Does not refer		Total
	Freq	%	Freq	%		Freq	%	Freq	%	
Poor	28	44.4	35	55.6	63(100)	41	65.1	22	34.9	63(100)
Fair	25	69.4	11	30.6	36(100)	32	88.9	4	11.1	36(100)
Good	6	66.7	3	33.3	9(100)	9	100	0	0.0	9(100)
**Total**	59	54.6	49	45.4	108(100)	82	75.9	26	24.1	108(100)
p-value*				0.042				0.005

* Fisher exact test

## Discussion

The majority of the TBAs in this study were aged 30 years and above, most of them were married and lived within the LGA in which they worked and over half were females. In addition, 69.5% of them had either primary or secondary education implying that most of them were literate. These socio-demographic findings were suited to the criteria put forward by Federal Ministry of Health for the selection of TBAs for training. [11] The level of awareness of HIV in this study was higher than that found among TBAs in other Nigerian studies. /5,12/ Awareness and knowledge of HIV/AIDS was found to be better than awareness and knowledge of PMTCT specifically. This was also discovered in studies carried out among TBAs and women (antenatal and postnatal patients) attending health facilities in Nigeria and other African nations [13-18].

Local government health workers were the most common source of information on HIV in this study followed by television and radio. Only 7.4% of respondents got HIV information from other TBAs. Similarly, in a study in Cross Rivers state, TBAs got their information on HIV mostly from government hospitals/health centers (34.2%) and only 8.6% got their information from other TBAs or traditional healers. [19] On the contrary, most (84.5%) of the TBAs studied in Ebonyi State got their information from their peers. [12] The fact that the TBAs in this study got their information on HIV mostly from health workers at their LGA may be an indication of the interaction between TBAs (who are recognized as PHC providers) and other PHC providers at the LGA.

Majority (98.1%) of the respondents correctly knew HIV to be an infection. Likewise, 92.6% of the TBAs interviewed in a study in Ikorodu, Lagos State knew HIV to be an infection unlike the TBAs surveyed in Cross River State where only 35.0% reported knowing what HIV means [19-20].

When asked to mention modes of transmission of HIV, only 20.4% of spontaneously mentioned transmission from an infected mother to child, however, when probed with a closed question, the proportions that were aware of MTCT increased to 90.7% and breastfeeding was most often mentioned as a possible period of MTCT. Awareness of MTCT of HIV was just as high among TBAs studied in Tanzania and Uganda; although only about half of them believed that transmission through breastfeeding was possible, the majority mentioned delivery, a finding contrary to that of this study [13].

Knowledge of ways to prevent MTCT of HIV was generally poor among the TBAs. The most common way mentioned was avoidance of breastfeeding, not surprising, as breastfeeding was the best-known period of MTCT among the respondents. Similarly, 50% of the Ugandan TBAs considered avoidance of breastfeeding as a method to prevent MTCT, less than a quarter of the Tanzanian TBAs considered caesarean section, drugs and avoidance of breastfeeding as possible means of prevention [13].

Only 8.3% of respondents had good level of knowledge about HIV and PMTCT. This inadequacy of knowledge and the use of traditional practices have been identified as causes of some deficiencies in the care that TBAs provide [21-22]. The females in this study had better knowledge than the males and this was found to be statistically significant (p = 0.014). This was in contrast to the findings among TBAs in Cross Rivers state where the males (73.7%) had better knowledge of HIV than the females (28.9%) [19].

This study did not demonstrate any statistically significant association between educational status and level of knowledge of respondents. It showed, however, that good level of knowledge influences some practices that could prevent MTCT. Among another group of TBAs and herbal practitioners studied in Lagos, more formally educated respondents had knowledge about sexual transmission of HIV compared to those with no formal education [5].

Several deficiencies were identified in the practices of TBAs in this study. The practices of HIV counseling and referral of all patients for HIV testing were poor. This could possibly be due to their low level of knowledge as those respondents with fair or good knowledge were the ones who mostly counseled or referred patients for testing. This trend is worrying especially as TBAs take about 60% of deliveries in Nigeria [5] and may indeed be the only source of health information to the patients they attend to.

Neglect of universal precautions is likely to put patients as well as TBAs at a risk of contracting HIV. Although majority (96.3%) of the respondents reported using gloves always, they were deficient in their sterilization practices, use of bleach to inactivate HIV in blood spills and instruments and wearing of protective clothing to take deliveries. These deficiencies were in consonance with results found from other TBAs studied in Nigeria. [5,12,19,20] Some of the respondents also mentioned the use of agbo, a local concoction which they drunk regularly and gave to pregnant women as a way to protect against HIV. The unscientific practices by the TBAs can encourage the spread of HIV by giving a false impression of being protected and therefore not employing more valid means of protection.

## Conclusion

The TBAs in this study had deficient knowledge and practice of PMTCT. This illustrates the need for periodic PMTCT training programs for TBAs and closer supervision in order for them to adopt safe and healthy practices to prevent HIV spread.

## Competing interests

The authors declared that they have no competing interests.

## Authors’ contributions

MB: contributed to the literature search, data collection, data analysis and the write-up of the manuscript. KO: contributed to the design of the study, data analysis and the write-up of the manuscript.

## Acknowledgments

The authors wish to acknowledge Dapo Asiyanbi and Nike Oluwo, the medical officers of health for Ajeromi-Ifelodun and Mushin LGAs respectively for their assistance during community entry and data collection.
